# *MICA ^∗^012:01* Allele Facilitates the Metastasis of KRAS-Mutant Colorectal Cancer

**DOI:** 10.3389/fgene.2020.00511

**Published:** 2020-05-26

**Authors:** Weifeng Ding, Yanyun Ma, Weifeng Zhu, Weilin Pu, Jianfeng Zhang, Fei Qian, Youlang Zhou, Yan Deng, Shicheng Guo, Jiucun Wang, Xiaodong Zhou

**Affiliations:** ^1^Department of Laboratory Medicine, Affiliated Hospital of Nantong University, Nantong, China; ^2^McGovern Medical School, The University of Texas, Houston, TX, United States; ^3^State Key Laboratory of Genetic Engineering, Collaborative Innovation Center for Genetics and Development, School of Life Sciences, Fudan University, Shanghai, China; ^4^Department of Biochemistry and Molecular Biology, College of Basic Medical Sciences, Nanchang University, Nanchang, China; ^5^Department of Gastroenterology, Affiliated Hospital of Nantong University, Nantong, China; ^6^Department of Gastrointestinal Surgery, Affiliated Hospital of Nantong University, Nantong, China; ^7^Department of Hand Surgery, The Hand Surgery Research Center, Affiliated Hospital of Nantong University, Nantong, China; ^8^Department of Ophthalmology of Children, The Second Affiliated Hospital of Nanchang University, Nanchang, China; ^9^Center for Precision Medicine Research, Marshfield Clinic Research Institute, Marshfield, WI, United States; ^10^Institute of Rheumatology, Immunology and Allergy, Fudan University, Shanghai, China; ^11^Human Phenome Institute, Fudan University, Shanghai, China

**Keywords:** MICA polymorphism, colorectal cancer, *MICA ^∗^012:01* allele, immunosurveillance, tumor immunity

## Abstract

Major histocompatibility complex (HLA) class I chain-related protein A (MICA) regulates immune surveillance through activation of NKG2D (natural killer group 2D) receptor. However, the genetic association, potential function, and predictive ability of MICA alleles with colorectal cancer (CRC) prognosis remain undefined. In this study, we characterized MICA alleles in tissue samples from 104 patients with CRC and 536 healthy controls and carried out genetic association studies by molecular and clinical CRC phenotypes. Preliminary sequence analysis revealed that *MICA ^∗^009:01* or *^∗^049* alleles were significantly decreased in patients with CRC (*p* = 0.0049), and further stratification analysis indicated that *MICA ^∗^012:01* allele was associated with patients with CRC and carrying KRAS codon 12 mutation (*p* = 0.027). The functional consequences of MICA alleles were examined via transfected CRC cell lines which showed that overexpression of *MICA ^∗^012:01* enhanced the proliferation, invasion, and metastatic phenotype of CRC. Preliminary analysis of disease-free survival time in patients with and without *MICA ^∗^012:01* suggest this allele may be predictive for poor prognosis of patients with KRAS codon 12 mutated CRC, as no somatic mutation of MICA gene was detected in CRC tumors compared to paracancerous tissues. Our study indicates that *MICA ^∗^012:01* allele is associated with KRAS-mutated CRC, facilitates CRC invasion and metastasis, and possibly reduces the survival of patients with KRAS-mutated CRC.

## Highlights

- Exon sequencing of *MICA* in clinical samples from 104 CRC biopsies and functional exploration of MICA alleles in CRC.
- *MICA ^∗^012:01* allele is associated with CRC subtype carrying KRAS codon 12 mutation [*p* = 0.027, odds ratio (OR) = 3.33].
- *MICA ^∗^009:01* or *^∗^049* allele was significantly decreased in patients with CRC (*p* = 0.0049, OR = 0.35).
- Overexpression of *MICA ^∗^012:01* allele enhanced the proliferation, invasion, and metastatic phenotype of CRC carrying KRAS mutation.
- Difference in MICA alleles may be associated with varied disease-free survival in KRAS-mutated CRC.

## Introduction

Colorectal cancer is the third most common cancer diagnosed and the second leading cause of cancer deaths in the United States (Siegel et al., 2019) and China (Chen W. et al., 2016). Since many factors contribute to CRC tumorigenesis, including genetic factors, epigenetic factors, race, gender, drug, type II diabetes, inflammatory bowel disease, and others (Dekker et al., 2019), CRC therapy has been a challenge for clinicians and researchers (Ferrari de Andrade et al., 2018; Wang et al., 2018; Courau et al., 2019). Although the pathogenesis of CRC is still unclear, it is believed that multiple genetic factors contribute to CRC susceptibility (Zarour et al., 2017; Dekker et al., 2019).

The human major histocompatibility complex (HLA) class I chain-related protein A (MICA) controls the immune process by interacting with its receptor, NKG2D (Zingoni et al., 2018) to regulate the activities of natural killer cells, γδ T-cells, αβ CD8 T-cells, and immunosuppressive CD4 T-cells (Choy and Phipps, 2010; Risti and Bicalho, 2017). The gene consists of 6 exons, in which exon 1 encodes the leader peptides, exons 2-4 encode three extracellular globular domains, exon 5 encodes the transmembrane (TM) domain, and exon 6 encodes the cytoplasmatic tail (Risti and Bicalho, 2017). MICA is one of the necessary negative regulators in cancer immunology (Glas et al., 2001), and both MICA and its corresponding receptor are highly expressed in carcinomas and inflammatory lesions (Zhou et al., 2014). High expression of MICA and NKG2D is strongly linked to tumor immunosurveillance (Groh et al., 2002; Choy and Phipps, 2010) while low levels of MICA are associated with a poor prognosis in patients receiving aggressive chemotherapy for CRC (Chen and Gyllensten, 2014).

MICA is a highly polymorphic gene (Glas et al., 2001) with about a total of 109 named alleles according to IPD-IMGT/HLA database^1^. In addition to these variants, *MICA* also contains tri-nucleotide microsatellite polymorphisms (GCT)n, which are designated as An, that start at codon 295 in the TM domain; a five GCT repetition can also coexist with a guanosine insertion that is designated as A5.1 (Zhou et al., 2014).

Although the associations of *MICA* polymorphisms with genetic predisposition to different cancer types have been investigated in candidate gene-based studies (Chen and Gyllensten, 2014; Ghadially et al., 2017), these previous studies predominantly focused on polymorphisms within the TM.

Therefore, the associations of MICA polymorphisms in exons 2–5 with CRC have not been reported. In this study, we investigated the genetic associations between molecular and clinical phenotypes of CRC and MICA alleles in a cohort of patients with CRC and healthy controls from Southern China and validated our results via functional analysis in CRC cell lines carrying KRAS driver gene mutation transfected with *MICA ^∗^012:01* and *^∗^008*. Preliminary analysis of disease-free survival time in patients with and without *MICA ^∗^012:01* may support the clinical relevance of this allele for CRC prognosis.

## Results

### MICA Alleles in CRC

**FIGURE 1:**
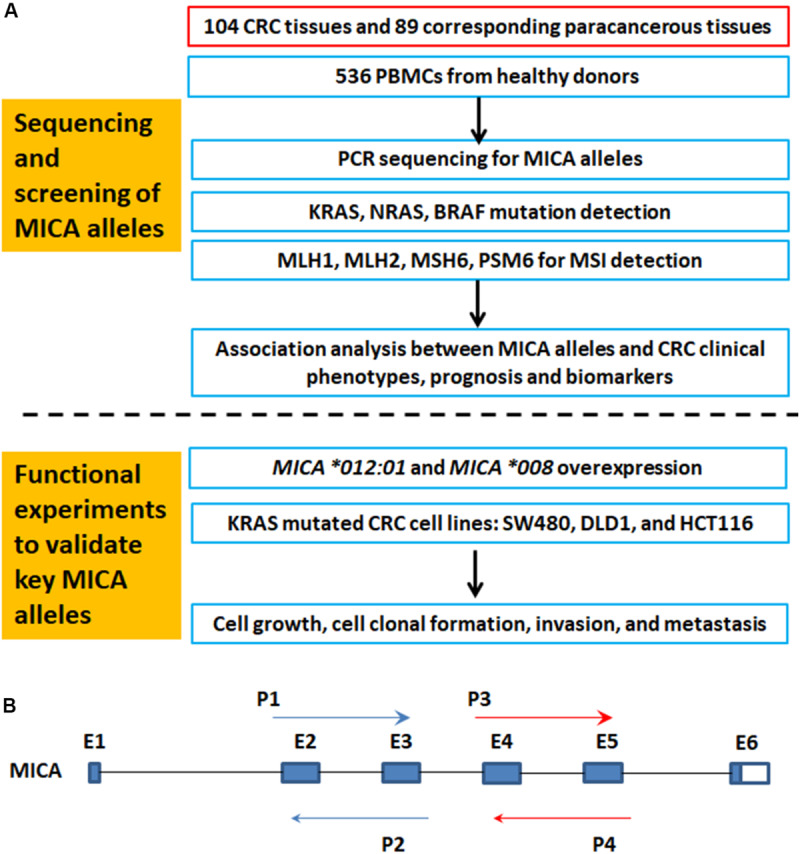
The flowchart of MICA genotyping and analysis of genetic association with CRC, as well as functional experiments. **(A)** Flowchart of study activities. **(B)** Exon-intron organization of MICA gene and primers for PCR sequencing. MICA, the human major histocompatibility complex class I chain-related gene A; P, primer; E, exon; CRC, colorectal cancer; MSI, microsatellite instability; KRAS, Kirsten rat sarcoma oncogene homolog; NRAS, neuroblastoma RAS viral oncogene homolog; BRAF, v-raf murine sarcoma oncogene homolog; MLH1, mutL homolog l; MSH2, mutS homolog 2; MSH6, mutS homolog 6; PMS2, PMS1 homolog 2.

Figure 1A is a flowchart of study procedures for MICA allele determination, association studies, and functional analysis in the context of CRC cell lines and patient samples. PCR sequencing analysis and comparison of base variations between 87 CRC tissue samples and corresponding paracancerous normal tissues did not detect any somatic mutations in *MICA* (Figure 1B). However, allele detection of the sampled population identified twelve *MICA* alleles in patients with CRC and fourteen alleles in the control group. Among these, *MICA ^∗^007:02* was only allele to occur in CRC samples while *MICA ^∗^009:02*, *^∗^018:01*, and *^∗^033* only occurred in controls. Frequency analysis revealed that *MICA ^∗^009:01* or *^∗^049* were significantly decreased in CRC compared to control samples [*p* = 0.0041 (odds ratio) OR = 0.35, Table 1].

**TABLE 1:** Comparison of MICA alleles between patient samples with CRC and healthy controls.

**Allele**	**CRC *n* = 104 (%)**	**Control *n* = 536 (%)**	**χ^2^**	***P* value**	**OR (95% CI)**
*002:01	22 (21.15)	164 (30.60)	**3.77**	**0.052**	0.61 (0.37–1.01)
*004	4 (3.85)	10 (1.87)	1.59	0.207	
*007:01	5 (4.81)	15 (2.80)	1.16	0.282	
*007:02	1 (0.96)	0 (0.00)	0.82	0.367	
*008	34 (32.69)	212 (39.55)	2.11	0.146	
*009:01 or *049	8 (7.69)	102 (19.03)	**7.87**	**0.0049**	0.35 (0.17–0.75)
*009:02	0 (0.00)	4 (0.75)	0.79	0.373	
*010:01	38 (36.54)	216 (40.30)	0.51	0.474	
*012:01	19 (18.27)	67 (12.50)	2.49	0.115	
*017	2 (1.92)	12 (2.24)	0.04	0.840	
*018:01	0 (0.00)	1 (0.19)	0.19	0.659	
*019	17 (16.35)	59 (11.01)	2.37	0.124	
*027	7 (6.73)	56 (10.45)	1.35	0.245	
*033	0 (0.00)	1 (0.19)	0.19	0.659	
*045	6 (5.77)	32 (5.97)	0.01	0.937	

*Bold values show the statistical significance.*

### Association of MICA Alleles With Driver Gene Mutations of CRC

Some driver genes’ mutations such as KRAS, NRAS, and BRAF can lead to CRC. Therefore, preliminary association analysis of *MICA* codon 295 polymorphisms and alleles to driver gene mutations, specifically KRAS codon 12 mutation, in CRC samples indicated that *MICA ^∗^012:01* was significantly associated with an increased OR for KRAS codon 12 mutation (*p* = 0.027, OR = 3.33, Table 2). MICA ^∗^A4 had a marginal but not significant association with the mutation of KRAS codon 12 (*p* = 0.105, OR = 2.25; Supplementary Table S1).

**TABLE 2:** Comparison of MICA alleles to driver gene mutations in CRC.

**Allele**	**Driver mutation *n* = 50 (%)**	**KRAS codon 12 *n* = 29 (%)**	**No mutation *n* = 52 (%)**
*002:01	15 (30.00)	10 (34.48)	10 (19.23)
*004	1 (2.00)	0 (00.00)	2 (3.85)
*007:01	4 (8.00)	1 (3.45)	1 (1.92)
*008	16 (32.00)	9 (31.03)	17 (32.69)
*009:01 or *049	6 (12.00)	4 (13.79)	3 (5.77)
*010:01	15 (30.00)	8 (27.59)	24 (46.15)
*012:01	12 (24.00)	**10 (34.48)^a^**	**7 (13.46)**
*017	0 (0.00)	0 (0.00)	2 (3.85)
*019	10 (20.00)	6 (20.69)	6 (11.54)
*027	2 (4.00)	2 (6.89)	5 (9.62)
*045	1 (2.00)	1 (3.45)	5 (9.62)

*^*a*^p = 0.027 KRAS codon 12 mutation vs. no mutation in CRC, Mantel-Haenszel stratification test χ^2^ = 4.90, OR = 3.33 (1.09–10.58). Bold values show the statistical significance.*

### Association of MICA Alleles With Molecular Typing of CRC

Stratification of CRC samples by CIN and MSI revealed that codon 295 *MICA* polymorphisms and *MICA ^∗^012:01* were associated with molecular typing of CRC; *MICA ^∗^012:01* was significantly increased in MSI and MSI-H type CRC (*p* = 0.0026, OR = 7.59 and *p* = 0.046, OR = 4.29, respectively; Table 3). Further analysis of MICA alleles in the context of *MICA* codon 295 polymorphism and CRC molecular subtype determined that A4 was significantly increased in MSI or MSI-H compared with CIN (*p* = 0.011 and *p* = 0.026, respectively; Supplementary Table S2). Similar results occurred when accounting for age and gender variations in the sampled population (Supplementary Table S3).

**TABLE 3:** Comparison of MICA alleles to molecular typing of CRC.

**Allele**	**MSI, *n* = 13 (%)**	**MSI-H, *n* = 10 (%)**	**CIN, *n* = 46 (%)**
*002:01	2 (15.38)	1 (10.00)	11 (23.91)
*004	0 (00.00)	0 (00.00)	2 (4.35)
*007:01	1 (7.69)	1 (10.00)	3 (6.52)
*008	5 (38.46)	4 (40.00)	15 (32.61)
*009:01 or *049	1 (7.69)	1 (10.00)	4 (8.69)
*010:01	4 (30.77)	4 (40.00)	20 (43.48)
*012:01	**6 (46.15)^a^**	**4 (40.00)^b^**	6 (13.04)
*017	0 (0.00)	0 (0.00)	2 (4.35)
*019	2 (15.38)	1 (10.00)	9 (19.57)
*027	1 (7.69)	1 (10.00)	4 (8.69)
*045	1 (7.69)	1 (10.00)	3 (6.52)

*^*a*^p = 0.0026 MSI CRC vs. CIN CRC, Mantel-Haenszel stratification test χ^2^ = 9.04, OR = 7.59 (1.72–35.91). ^*b*^p = 0.046 MSI-H CRC vs. CIN CRC, Mantel-Haenszel stratification test χ^2^ = 4.00, OR = 4.29 (0.85–21.02). Bold values show the statistical significance.*

### MICA Alleles Were Associated With Clinical Phenotypes of CRC

We conducted further association studies of *MICA* codon 295 polymorphisms and MICA alleles with tumor characteristics such as differential status, UICC stage, tumor size, invasion depth, lymph nodes metastasis, and gross classification, and discovered that A5.1 was significantly increased in highly differentiated CRC compared to that in medium state (*p* = 0.019; Supplementary Table S4). Other notable, though not statistically significant, associations with *MICA* codon 295 polymorphisms was the presence of A5.1 in medium compared to low differentiated CRC (*p* = 0.07) and decreased A6 in protruded CRC compared to ulcerated CRC (*p* = 0.089, Supplementary Table S4). Similar results occurred when adjusting for age and gender variations in the sampled population (Supplementary Table S5).

In the context of MICA alleles, *MICA ^∗^045* was significantly increased in the protruded type of CRC (*p* = 0.0028, Supplementary Table S6) while *MICA ^∗^027* was notably increased in UICC stage III/IV of CRC compared with stage I/II samples (*p* = 0.044, Supplementary Table S6). Though not statistically significant, *MICA ^∗^019* had low association with tumor size and invasion depth of CRC (*p* = 0.064 and *p* = 0.096, respectively, Supplementary Table S6) as did *MICA ^∗^008* to the distance metastasis and differential degree of CRC (*p* = 0.053 and *p* = 0.071, respectively, Supplementary Table S6).

### Association of MICA Alleles With Protein Expression of Immune Checkpoint PD-L1 and Tumor Biomarkers CEA, CA19-9, and CYFRA21-1

Programmed Death-Ligand 1 expression is a key factor in cancer immune therapy response. CEA, CA19-9, and CYFRA21-1 are widely used biomarkers in clinical practice for CRC diagnosis and progression. Though the expression of PD-L1 was not significantly associated with MICA codon 295 (Supplementary Table S7) there may be an association between MICA alleles *^∗^009:01* or *^∗^049* and PD-L1 expression (*p* = 0.094). Moreover, *MICA ^∗^010:01* was decreased in patients with positive CA19-9 (*p* = 0.077, OR = 0.23; Supplementary Table S8).

### Overexpression of *MICA ^∗^012:01* Significantly Enhanced the Proliferative Ability of KRAS Mutated CRC

**FIGURE 2:**
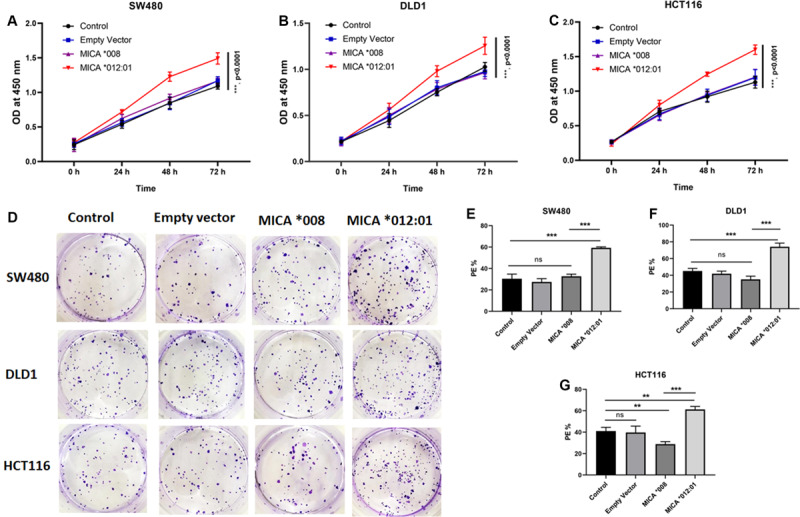
Cell proliferation and colony forming assays of KRAS mutated CRC cell lines with expression of MICA *012:01 or MICA *008 allele. Growth curves were examined by CCK-8 assay in **(A)** SW480 cells, **(B)** DLD cells, and **(C)** HCT116 cells. **(D)** The proliferative capability of MICA alleles was determined by cell clone formation experiments with corresponding histograms shown in **(E–G)**. Cell colony-formatting efficiency was calculated as: PE (Planting efficiency)% = Cell clone/Inoculation cell number × 100%. Control, untransfected cells; Empty vector, cells transfected with pEGFP-C3 plasmid vector; MICA *008, cells transfected with pEGFP-MICA *008 recombination vector; MICA *012:01, cells transfected with pEGFP-MICA *012:01 recombination vector. ns, no significance; *p < 0.05; **p < 0.01; ***p < 0.001.

Since MICA alleles are associated with molecular and clinical phenotypes of CRC, we hypothesized that *MICA ^∗^012:01* and *MICA ^∗^008* alleles have marked functional differences. Therefore, CRC cell lines SW480, HCT116, and DLD1, which carry KRAS mutations, were transfected with *MICA ^∗^012:01* and *MICA ^∗^008* overexpression plasmid vectors. Through a CCK-8-based proliferation assay, we found that the *MICA ^∗^012:01* overexpressed group had a higher proliferation rate than *MICA ^∗^008* overexpressed group, untransfected control, and empty plasmid groups in SW480, HCT116, and DLD1 cells (Figures 2A–C; all *p* < 0.0001). To characterize the proliferative behavior of MICA overexpressed cells, we performed cell colony formation experiments and determined that overexpression of *MICA ^∗^012:01* significantly enhanced the colony formation ability of proliferating KRAS mutated CRC cell lines (Figures 2D–G; *p* = 0.0004, 0.0007, and 0.0046, respectively).

### *MICA ^∗^012:01* Allele Significantly Enhanced the Invasion and Metastatic Ability of KRAS Mutated CRC Cells

**FIGURE 3:**
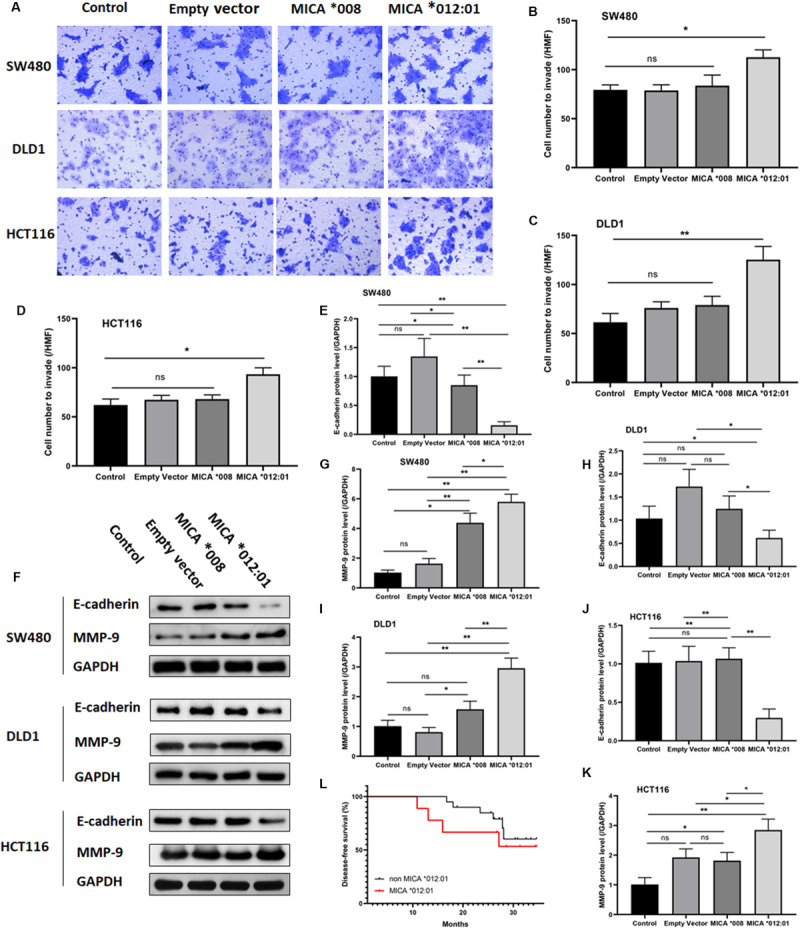
The invasive and metastatic properties of KRAS mutated CRC cell lines transfected with MICA *012:01 or MICA *008 allele. The invasive capability of MICA allele overexpressing CRC cell clines was evaluated by transwell assay in **(A)** SW480 cells, DLD cells, and HCT116 cells. HMF, high magnification field (400 fold) with corresponding histograms **(B–D)**. ns, no significance; *p < 0.05; **p < 0.01. **(E)** Relative amounts of total MMP-9 and E-cadherin protein levels were analyzed by Western blotting experiments. Representative blots are shown. **(F–K)** Corresponding histograms of the results are shown. ns, no significance; *p < 0.05; **p < 0.01. **(L)** The disease-free survival curve of MICA *012:01 and non-MICA *012:01 alleles in CRC patients carrying KRAS codon 12 mutation. Control, untransfected cells; Empty vector, cells transfected with pEGFP-C3 plasmid vector; MICA *008, cells transfected with pEGFP-MICA *008 recombination vector; MICA *012:01, cells transfected with pEGFP-MICA *012:01 recombination vector.

To further determine whether overexpression of *MICA ^∗^012:01* allele could exacerbate the malignant phenotype of CRC, we also assayed the invasive and metastatic capability of CRC cell lines via transwell and Western blot experiments. As shown in Figures 3A–D, cells transfected with *MICA ^∗^012:01* were markedly more invasive than those overexpressing *MICA ^∗^008*, untransfected control, and empty plasmid groups in SW480, DLD1, and HCT116 cells (*p* = 0.0188, 0.0076, and 0.0050, respectively). Western blot analysis of extracellular matrix degrading enzyme matrix metalloproteinase-9 (MMP-9) and cell-cell adhesion protein E-cadherin revealed notably increased MMP-9 expression in the MICA ^∗^012:01 overexpressed group compared to *MICA ^∗^008*, and transfection control groups in SW480, DLD1, and HCT116 cells (Figures 3E,G,I,K). In contrast, the expression level of E-cadherin was significantly decreased in the *MICA ^∗^012:01* overexpressed group than the remaining groups in all cell lines tested (Figures 3E,F,H,J). Taken together, our data demonstrate that overexpression of *MICA ^∗^012:01* allele significantly enhanced the invasiveness and metastatic ability of CRC cell lines carrying KRAS mutations.

To validate the functional role of *MICA ^∗^012:01* in CRC malignancy, we analyzed the disease-free survival time (DFS) of patients with CRC that carry the KRAS codon 12 mutation and contain *MICA ^∗^012:01* to those that do not have *MICA ^∗^012:01*. Kaplan Meyer survival curve analysis did not reveal a statistically significant difference in DFS by the presence of *MICA ^∗^012:01* (*p* = 0.103) though we note that our sample size is underpowered for this analysis. However, CRC patients with *MICA ^∗^012:01* had a tendency to experience relapse or metastasis in the first 20 months after the surgery than those with no *MICA ^∗^012:01*. Therefore, *MICA ^∗^012:01* allele expression may be a predictive marker for poor prognosis in patients with KRAS codon 12 mutated CRC.

## Discussion

MICA is commonly expressed in tumor cells as a tumor-associated antigen that works in tandem with NKG2D receptor to regulate immunosurveillance (Groh et al., 2002; Choy and Phipps, 2010). Analysis of *MICA* polymorphisms indicates that MICA is strongly associated with immunologic escape of tumors by MICA shedding or other mechanisms (Groh et al., 2002; Chen and Gyllensten, 2014; Ji et al., 2015). Our genetic association studies herein indicate that *MICA ^∗^009:01* or *^∗^049* were associated with decreased frequency of certain CRC subtypes, and *MICA ^∗^012:01* was increased in patient samples carrying KRAS codon 12 mutation. Functional studies of SW480, DLD1, and HCT116 cell lines transfected with MICA alleles indicated that *MICA ^∗^012:01* allele enhanced the malignant phenotype of CRC in cell lines that express KRAS mutation. Examination of tumor tissues did not show any somatic mutations in exons 2–5 of the MICA gene which may indicate that certain MICA alleles are associated with increased susceptibility to CRC progression.

In addition to specific MICA alleles, polymorphisms in the TM region of the gene, especially in ^∗^An, also showed specific associations in Chinese patients with CRC. The frequency of *MICA ^∗^A5.1* was decreased in medium or low differention state in patient samples compared to those with highly differented CRC (Supplementary Tables S4, S5). On the other hand, the frequencies of *MICA ^∗^A6* and *^∗^A9* that carry relatively longer repetitions of GCT are significantly less frequent in Chinese patients with CRC, and *MICA ^∗^A4*, which carries the shortest repetition of GCT, was increased in Chinese patients with CRC carrying a KRAS mutation at codon 12 (Supplementary Table S1). These results are in contrast to Kopp et al. (2009), who previously reported that MICA TM polymorphisms had no difference in the normal colonic mucosa and colorectal tumor tissue with MSI and KRAS codon 12 mutation subtypes in a European population (Kopp et al., 2009). This inconsistency may be due to variation in genotype distribution of *MICA* among different races. For instance, *MICA ^∗^008:01*, *^∗^004*, and *^∗^009:02* are the most common alleles in a French population (Carapito et al., 2017), yet according to Zarour et al. (2017) *MICA ^∗^002:01*, *^∗^008:01* or *04*, and *^∗^010:01* are the most prevalent alleles in the Chinese population. However, it is worth noting that *MICA* codon 295 polymorphism *^∗^A5.1* results in a premature stop codon that leads to a break in TM region of MICA to produce a soluble MICA (sMICA) (Zhou et al., 2014) that interacts with NKG2D, resulting in NKG2D internalization and subsequent suppression or impairment of NKG2D-mediated immune response (Groh et al., 2002) that may alter susceptibility to CRC development. Whether the GCT repetition length can affect stability and/or functional changes of MICA is still unknown.

MMP-9 is a matrix remodeling enzyme expressed in alveolar macrophages, granulocytes and mast cells that plays essential roles in leukocyte migration and tumor cell metastasis among others (Tsai et al., 2014; Chen S. et al., 2016; Huttlin et al., 2017; Yang et al., 2017). In contrast, E-cadherin, which belongs to the Ca^2+^ dependent cell adhesion molecule family (Guilford et al., 1998), regulates cell-cell adhesions, mobility, and proliferation of epithelial cells and is a potent invasive suppressor for tumor cells (Ma et al., 2010; Satow et al., 2014; Indra et al., 2018). Our cell line based functional studies indicate that overexpression of *MICA ^∗^012:01* correlated with increased expression of MMP-9 and corresponding down-regulation of E-cadherin (Figure 3E). Moreover, in sharp contrast to the common allele *MICA ^∗^008*, overexpression of *MICA ^∗^012:01* allele significantly exacerbated the malignant phenotype of KRAS mutated CRC similar to results from other groups (Simanshu et al., 2017; Janes et al., 2018; Li et al., 2018; Brandt et al., 2019; Serebriiskii et al., 2019; Figures 2, 3).

Analysis of DFS in clinical patients with and without *MICA ^∗^012:01* did not reveal a statistically significant difference in DFS though it should be noted that the sample size was underpowered to detect a significant difference for a low effect allele. Future studies will incorporate additional data from more patient samples.

Taken together, we provide evidence that *MICA ^∗^012:01*, *^∗^009:01* or *^∗^049*, and *MICA ^∗^A4* are important genetic factors associated with CRC, CRC with KRAS mutation, and MSI mutation subtypes of CRC in a Chinese population. Overexpression of *MICA ^∗^012:01* allele enhanced the malignant phenotype of CRC with KRAS mutation specifically proliferation, invasion, and metastasis in cultured CRC cell line which may suggest that *MICA ^∗^012:01* enhances the metastatic potential of CRC by interacting with the NKG2D to evade immune surveillance.

## Materials and Methods

### CRC and Control Samples

A total of 104 patients with CRC were enrolled from the Department of General Surgery at the Affiliated Hospital of Nantong University, Jiangsu Province, China. Tumors were classified according to TNM/UICC criteria (Provenzale et al., 2015) following histopathological examination. All patients were enrolled under the screening and diagnosis criteria of CRC (Provenzale et al., 2015). A total of 536 control samples (whole blood) were obtained from a study project of Chinese population genetics in Fudan University, Shanghai, China (Zhou et al., 2014), and these patients were enrolled from clinics and hospitals in southern cities of China. Samples were collected from individuals of Southern Han Chinese ancestry. The average ages of CRC patients and controls were 66 and 46, respectively and gender ratios varied between groups from 69% men and 31% women with CRC, and 51% men versus 49% women for control samples. Postoperative follow-up was performed according to recommendations from the Chinese Society of Clinical Oncology (CSCO).

### MICA Sequencing and Genotyping

Genomic DNA was extracted from peripheral blood cells from control samples and from intestinal epithelial cells from colorectal tumors and paracancerous normal intestine tissue that is > 10 sm distant from the edge of cancer, respectively. *MICA* was genotyped by PCR sequencing of exons 2, 3, 4, and 5 using bidirectional Sanger sequencing methods (Zhou et al., 2014). Sequencing data were analyzed by Chromas 2.4.1 software (technelysium, Au). Allelic genotypes of *MICA* in each sample were obtained according to reference sequence of specific MICA alleles^2^. For quality control in DNA typing, all sequencing processes were performed using robotic automation system to minimize sample mislabeling and misplacing. We also had blind duplicates in each sample plate.

### Screening for Tumor Microsatellite Instability and KRAS, NRAS, and BRAF Oncogene Mutations

Analysis of tumor MSI was performed using fluorescence *in situ* hybridization (FISH) to detect MLH1, MSH2, MSH6, and PMS2 (Sepulveda et al., 2017). The positive expression of all four genes was judged as MSS or CIN while three or less gene positive expression was considered as MSI.

Driver mutations in codons 12, 13, 59, 61, 117, and 146 of KRAS and NRAS genes, and the mutation in codon 600 of BRAF gene (Sepulveda et al., 2017) in patient samples were analyzed by FastTarget next-generation sequencing (Zhang et al., 2018).

### Cell Lines

Colorectal cancer cell lines SW480, DLD1, and HCT116 were purchased from American Type Culture Collection (ATCC; Manassas, VA, United States). SW480 and DLD1 cells were cultured with Dulbecco’s modified eagle medium (DMEM) with high glucose medium (Hyclone, Logan, UT, United States), HCT116 cells were cultivated with McCoy’s 5A (Thermo Fisher Scientific, United States) supplemented with 10% fetal bovine serum (FBS) (Gibco, United States) at 37^o^C in 5% CO_2_. SW480 cells carry KRAS G12V mutation and MSS (Ahmed et al., 2013) while DLD1 and HCT116 cells carry KRAS G13D mutation and MSI (Ahmed et al., 2013).

### Plasmid Vectors and Transfection

pEGFP-C3 plasmid vector was developed and maintained by our laboratory. The full-length cDNA of *MICA ^∗^012:01* and *^∗^008* were chemically synthesized by RuiMian Bio (Shanghai, China) and inserted into thepEGFP-C3 plasmid after digestion with restriction enzymes *Kpn*I and *Eco*RI (Fermantas lnc., Burlington, ON, Canada). The plasmid was then ligated to form pEGFP-*MICA ^∗^012* and pEGFP-*MICA ^∗^008* and transfected into CRC cells by DNA transfection agent Entranster^TM^ H4000 (Engreen, Beijing, China).

### Cell Proliferation Assay With CCK-8 Detection

Cell proliferation assays were performed using Cell Counting Kit-8 (CCK-8; Keygen, China) according to the manufacturer’s instructions. Briefly, SW480, DLD1, and HCT116 cells were seeded in 96-well plates in DMEM or McCoy’s 5A with 0.5% FBS at a density of 2 × 10^3^ cells/well and transfected with pEGFP-*MICA ^∗^012* and control. At 24, 48, 72, and 96 h, the optical density at 450 nm wavelength, which correlates to the number of viable cells, was measured and the results expressed as mean of OD450 ± SEM.

### Detection of Cell Colony Formation Ability

Cells were seeded in a 24-well ultra-low attachment round bottom plate (Excell, United States) at a density of 300 cells per well in 10% FBS DMEM/McCoy’s 5A medium supplemented with pEGFP-C3 plasmid, pEGFP-*MICA ^∗^008*, or pEGFP-*MICA ^∗^012* transfection, respectively, and cultured for 8 days. Cultured cells were washed with phosphate buffered saline (PBS), fixed with 200 μL absolute methanol for 10 min, and then stained with Giemsa dye. The number of violet cell spheres was counted manually. Cell colony-formatting efficiency was calculated as: PE (Planting efficiency)% = Cell clone/Inoculation cell number × 100%.

### Cell Invasion Assay With Transwell Experiment

The cell invasion assay was performed using invasion chambers consisting of a 12-well tissue culture plate with 24-well sized inserts (Corning, NY, United States). Cell suspensions (0.5 × 10^6^ cells/mL) were transfected with pEGFP-*MICA ^∗^012:01*, pEGFP-*MICA ^∗^008*, or medium alone were added to the interior of the inserts with 300 μL serum-free media and 500 μL of media containing 10% FBS as a chemoattractant to the lower chamber and incubated for 72 h. Invasive cells on the lower surface of the membrane were stained by crystal violet for 10 min and imaged under the high magnification field (HMF, 400 fold) of the microscope.

### Western Blotting (WB) Analysis

Total protein was extracted using radioimmunoprecipitation (RIPA) lysis buffer (Beyotime, China) and quantitated by Bicinchoninic Acid (BCA) protein assay (Beyotime, China). Protein samples were separated by 10% sodium dodecyl sulfate/polyacrylamide gel electrophoresis (SDS/PAGE) and transferred to 0.45 μm polyvinylidene fluoride (PVDF) membrane (Millipore, United States). Following blocking with 5% non-fat milk (Beyotime, China), membranes were incubated with anti-E-cadherin (CST, United States) and anti-MMP-9 antibodies (CST, United States) overnight at 4°C and incubated with HRP-conjugated secondary antibodies (Abcam, United States) for 1 hr at room temperature. Protein bands were detected using enhanced chemiluminescence (ECL) detection kit (Beyotime, China) and Amersham Imager 600 system (GE Healthcare, United States). GAPDH was used as the loading control.

### Statistical Analysis

Correlations of MICA alleles with histopathological, genetic, and other molecular parameters were evaluated. Parameters include tumor invasion, lymph node involvement, histological type, presence of distant metastasis, UICC stages, MSI, driver gene mutation, immune checkpoint PD-L1, and diagnostic biomarkers. Exact *p*-values were obtained via Fisher’s test or Mantel-Haenszel stratification test from 2 × 2 tables of allele counts. 3-year disease-free survival times were calculated by Kaplan-Meier curves and compared by the log rank test. Two-tailed *t* tests and ANOVA were used to the functional experiments analysis. A *p*-value of less than 0.05 was used to indicate statistical significance. Statistical analysis was performed using SPSS statistical software (SPSS version 20.0).

## Data Availability Statement

The datasets generated during the current study available from the corresponding authors on reasonable request.

## Ethics Statement

The study was approved by the Ethics Committee of Affiliated Hospital of Nantong University, and patients’ informed consent was obtained.

## Author Contributions

All authors contributed to conception and design of study, analysis and interpretation of data, drafting the article and revising it critically for important intellectual content, and final approval of the version to be published.

## Conflict of Interest

The authors declare that the research was conducted in the absence of any commercial or financial relationships that could be construed as a potential conflict of interest.

The reviewer JX declared a shared affiliation, though no other collaboration, with one of the authors, XZ, to the handling Editor.

## Footnotes

1 http://www.ebi.ac.uk/imgt/hla/align.html

2 http://www.ebi.au.uk/imgt/hla

## Abbreviations

AS: ankylosing spondylitis
BRAF: v-raf murine sarcoma oncogene homolog
CA19-9: carbohydrate antigen 19-9
CIN: chromatin instability
CRC: colorectal cancer
KRAS: Kirsten rat sarcoma oncogene homolog
MICA: human major histocompatibility complex (HLA) class I chain-related protein A
MLH1: mutL Homolog
MSH2: mutS Homolog 2
MSH6: mutS Homolog 6
MSI: microsatellite instability
MSI-H: high microsatellite instability
MSS: microsatellite stability
NKG2D: natural killer group 2D
NRAS: neuroblastoma RAS viral oncogene homolog
OR: odds ratio
PD-L1: programmed death-ligand 1
PMS2: PMS1 Homolog 2
TM: transmembrane domain
TNM: tumor node metastasis
UICC: Union for International Cancer Control.
