# Supplementary material for: MICA ∗012:01 Allele Facilitates the Metastasis of KRAS-Mutant Colorectal Cancer
Source: Front Genet. 2020 May 26;11:511. doi: 10.3389/fgene.2020.00511 (PMC7264413; doi:10.3389/fgene.2020.00511)
Supplement: Supplementary file 1 [file Data_Sheet_1.docx]

**Supplementary files**

**Supplementary Results**

**Supplemental Table S1:** **Comparison of codon 295 polymorphisms of *MICA* to driver gene mutation in patients with CRC**

| Mutation Type | A4 (%) | A5 (%) | A5.1 (%) | A6 (%) | A9 (%) |
| --- | --- | --- | --- | --- | --- |
| Driver mutation (n=50) | 17 (34.00) | 25 (50.00) | 16 (32.00) | 8 (16.00) | 15 (30.00) |
| KRAS codon 12 (n=29) | **11 (37.93)^a^** | 14 (48.28) | 9 (31.03) | 5 (17.24) | 10 (34.48) |
| No mutation (n=52) | 11 (21.15) | 31 (59.62) | 21 (40.38) | 8 (15.38) | 12 (23.08) |

a, p= 0.105 KRAS codon 12 mutation vs no mutation in CRC, Mantel-Haenszel stratification test χ^2^=2.62, OR=2.25 (0.81-6.29)

**Supplemental Table S2: Comparison of codon 295 polymorphisms of *MICA* to molecular typing of patients with CRC**

|  | A4 (%) | A5 (%) | A5.1 (%) | A6 (%) | A9 (%) |
| --- | --- | --- | --- | --- | --- |
| MSI (n=13) | **8 (61.54)^a^** | 6 (46.15) | 5 (38.46) | 1 (7.69) | 2 (15.38) |
| MSI-H (n=10) | **6 (60.00)^b^** | 6 (60.00) | 5 (50.00) | 1 (10.00) | 1 (10.00) |
| CIN (n=46) | 11 (23.91) | 26 (56.52) | 16 (34.78) | 8 (17.39) | 13 (28.26) |

a, p= 0.011 MSI CRC vs CIN CRC, Mantel-Haenszel stratification test χ^2^=6.46, OR=4.93 (1.33-19.83)

b, p= 0.026 MSI-H CRC vs CIN CRC, Mantel-Haenszel stratification test χ^2^=4.97, OR=4.62 (1.07-21.73)

**Supplemental Table S3: Comparison of the presence of MICA alleles between patients with CRC and healthy controls after adjusting for age and gender**

| Allele | CRC patients n=104 (%) | Controls  n=536 (%) | χ^2^ | P value | OR (95% CI) |
| --- | --- | --- | --- | --- | --- |
| *002:01 | 22 (21.15) | 164 (30.60) | 3.23 | 0.068 | 0.61 (0.35-1.03) |
| *004 | 4 (3.85) | 10 (1.87) |  |  |  |
| *007:01 | 5 (4.81) | 15 (2.80) |  |  |  |
| *007:02 | 1 (0.96) | 0 (0.00) |  |  |  |
| *008 | 34 (32.69) | 212 (39.55) |  |  |  |
| *009:01 or *049 | 8 (7.69) | 102 (19.03) | 7.09 | 0.0077 | 0.35 (0.14-0.76) |
| *009:02 | 0 (0.00) | 4 (0.75) |  |  |  |
| *010:01 | 38 (36.54) | 216 (40.30) |  |  |  |
| *012:01 | 19 (18.27) | 67 (12.50) |  |  |  |
| *017 | 2 (1.92) | 12 (2.24) |  |  |  |
| *018:01 | 0 (0.00) | 1 (0.19) |  |  |  |
| *019 | 17 (16.35) | 59 (11.01) |  |  |  |
| *027 | 7 (6.73) | 56 (10.45) |  |  |  |
| *033 | 0 (0.00) | 1 (0.19) |  |  |  |
| *045 | 6 (5.77) | 32 (5.97) |  |  |  |

**Supplemental Table S4: Comparison of codon 295 polymorphisms of *MICA* to the clinical phenotype of patients with CRC**

|  | A4 (%) | A5 (%) | A5.1 (%) | A6 (%) | A9 (%) |
| --- | --- | --- | --- | --- | --- |
| Gross classification |  |  |  |  |  |
| Ulcerated (n=72) | 18 (25.00) | 40 (55.56) | 24 (33.33) | **14 (19.44)** | 17 (23.61) |
| Protruded (n=30) | 10 (33.33) | 15 (50.00) | 12 (40.00) | **2 (6.67) ^a^** | 10 (33.33) |
| Tumor size |  |  |  |  |  |
| ≤3 cm (n= 38) | 10 (26.32) | 17 (44.74) | 13 (34.21) | 8 (21.05) | 11 (28.95) |
| >3 cm (n=66) | 18 (27.27) | 40 (60.61) | 25 (37.88) | 9 (13.64) | 16 (24.24) |
| Invasion depth |  |  |  |  |  |
| T1+T2 (n=26) | 5 (19.23) | 15 (57.69) | 11 (42.31) | 4 (15.38) | 8 (30.77) |
| T3+T4 (n=76) | 23 (30.26) | 41 (54.95) | 26 (34.21) | 12 (15.79) | 19 (25.00) |
| Lymph node involvement |  |  |  |  |  |
| N0 (n=57) | 16 (28.07) | 30 (52.63) | 22 (38.60) | 9 (15.79) | 16 (28.07) |
| N1-3 (n=45) | 12 (26.67) | 26 (57.78) | 15 (33.33) | 7 (15.56) | 11 (24.44) |
| Distance metastasis |  |  |  |  |  |
| M0 (n=93) | 26 (27.96) | 52 (55.91) | 32 (34.41) | 16 (17.20) | 26 (27.96) |
| M1 (n=11) | 2 (18.18) | 5 (45.45) | 5 (45.45) | 1 (9.09) | 1 (9.09) |
| UICC stage |  |  |  |  |  |
| I/II (n=53) | 16 (30.19) | 29 (54.72) | 19 (35.85) | 9 (16.98) | 15 (28.30) |
| III/IV (n=51) | 12 (23.53) | 28 (54.91) | 18 (35.29) | 8 (15.69) | 12 (23.53) |
| Differentiation degree |  |  |  |  |  |
| High (n=8) | 3 (37.50) | 3 (37.50) | **6 (75.00)** | 1 (12.50) | 2 (25.00) |
| Medium (n=81) | 21 (25.93) | 48 (59.26) | **27 (33.33) ^b^** | 12 (14.81) | 23 (28.39) |
| Low (n=14) | 3 (21.43) | 6 (42.86) | **5 (35.71) ^c^** | 4 (28.57) | 2 (14.29) |

a, p= 0.089 ulcerated CRC vs protruded CRC, OR=3.379

b, p= 0.019 High differential CRC vs medium differential CRC, OR=6.00

c, p= 0.07 High differential CRC vs low differential CRC, OR=5.40

**Supplemental Table S5: Comparison of codon 295 polymorphisms of *MICA* to the clinical phenotype of patients with CRC after adjusting for age and gender**

|  | A4 (%) | A5 (%) | A5.1 (%) | A6 (%) | A9 (%) |
| --- | --- | --- | --- | --- | --- |
| Gross classification |  |  |  |  |  |
| Ulcerated (n=72) | 18 (25.00) | 40 (55.56) | 24 (33.33) | **14 (19.44)** | 17 (23.61) |
| Protruded (n=30) | 10 (33.33) | 15 (50.00) | 12 (40.00) | **2 (6.67)^a^** | 10 (33.33) |
| Tumor size |  |  |  |  |  |
| ≤3 cm (n= 38) | 10 (26.32) | 17 (44.74) | 13 (34.21) | 8 (21.05) | 11 (28.95) |
| >3 cm (n=66) | 18 (27.27) | 40 (60.61) | 25 (37.88) | 9 (13.64) | 16 (24.24) |
| Invasion depth |  |  |  |  |  |
| T1+T2 (n=26) | 5 (19.23) | 15 (57.69) | 11 (42.31) | 4 (15.38) | 8 (30.77) |
| T3+T4 (n=76) | 23 (30.26) | 41 (54.95) | 26 (34.21) | 12 (15.79) | 19 (25.00) |
| Lymph node involvement |  |  |  |  |  |
| N0 (n=57) | 16 (28.07) | 30 (52.63) | 22 (38.60) | 9 (15.79) | 16 (28.07) |
| N1-3 (n=45) | 12 (26.67) | 26 (57.78) | 15 (33.33) | 7 (15.56) | 11 (24.44) |
| Distance metastasis |  |  |  |  |  |
| M0 (n=93) | 26 (27.96) | 52 (55.91) | 32 (34.41) | 16 (17.20) | 26 (27.96) |
| M1 (n=11) | 2 (18.18) | 5 (45.45) | 5 (45.45) | 1 (9.09) | 1 (9.09) |
| UICC stage |  |  |  |  |  |
| I/II (n=53) | 16 (30.19) | 29 (54.72) | 19 (35.85) | 9 (16.98) | 15 (28.30) |
| III/IV (n=51) | 12 (23.53) | 28 (54.91) | 18 (35.29) | 8 (15.69) | 12 (23.53) |
| Differentiation degree |  |  |  |  |  |
| High (n=8) | 3 (37.50) | 3 (37.50) | **6 (75.00)** | 1 (12.50) | 2 (25.00) |
| Medium (n=81) | 21 (25.93) | 48 (59.26) | **27 (33.33)^b^** | 12 (14.81) | 23 (28.39) |
| Low (n=14) | 3 (21.43) | 6 (42.86) | **5 (35.71)^c^** | 4 (28.57) | 2 (14.29) |

a, p= 0.107 ulcerated CRC vs protruded CRC, OR=3.346

b, p= 0.051 High differential CRC vs medium differential CRC, OR=5.87

c, p= 0.18 High differential CRC vs low differential CRC, OR=4.96

**Supplemental Table S6: Comparison of MICA alleles to the clinical phenotype of patients with CRC**

|  | *002:01 (%) | *004 (%) | *007:01 (%) | *008 (%) | *009:01 or *049 (%) | | | *010:01 (%) | | *012:01 (%) | | *017 (%) | | *019 (%) | | *027 (%) | | | | | *045  (%) | | | | |
| --- | --- | --- | --- | --- | --- | --- | --- | --- | --- | --- | --- | --- | --- | --- | --- | --- | --- | --- | --- | --- | --- | --- | --- | --- | --- |
| Gross classification |  |  |  |  |  | | |  | |  | |  | |  | |  | | | | |  | | | | |
| Ulcerated  (n=72) | 16 (22.2) | 4 (5.6) | 3 (4.2) | 20 (27.8) | 8  (11.1) | | | 27 (37.5) | | 12 (16.7) | | 1 (1.4) | | 12 (16.7) | | 6  (8.3) | | | | | **1**  **(1.4)** | | | | |
| Protruded  (n=30) | 9 (30.0) | 0 (0.0) | 2 (6.7) | 12 (40.0) | 2  (6.7) | | | 11 (36.7) | | 7  (23.3) | | 1 (3.3) | | 4  (13.3) | | 1  (3.3) | | | | | **5**  **(16.7) ^a^** | | | | |
| Tumor size |  |  |  |  |  | | |  | |  | |  | |  | |  | |  | | | | |  |  |  |
| ≤3 cm (n= 38) | 10 (26.3) | 2 (5.3) | 1 (2.6) | 11  (29) | 4  (10.5) | | | 13 (34.2) | | 8  (21.1) | | 1 (2.6) | | **3**  **(7.9)** | | 3  (7.9) | | | | | | 2  (5.3) | | |  |
| >3 cm (n=66) | 15 (22.7) | 2 (3.0) | 4 (6.1) | 23 (34.9) | 6  (9.09) | | | 26 (39.4) | | 11 (16.7) | | 1 (1.5) | | **14 (21.2) ^b^** | | 4 (6.1) | | 4  (6.06) | | | | |  |  |  |
| Invasion depth |  |  |  |  |  | | |  | |  | |  | |  | |  | |  | | | | |  |  |  |
| T1+T2 (n=26) | 7 (26.9) | 0 (0.0) | 2 (7.7) | 9 (34.6) | 1  (3.9) | | | 8 (30.8) | | 3  (11.5) | | 1 (3.9) | | **7**  **(26.9)** | | 2 (7.7) | | 2  (7.7) | | | | |  |  |  |
| T3+T4 (n=76) | 18 (23.7) | 4 (5.3) | 3  (4) | 24 (31.6) | 8  (10.5) | | | 30 (39.5) | | 16 (21.1) | | 1 (1.3) | | **10 (13.2) ^c^** | | 5 (6.6) | | 4  (5.3) | | | | |  |  |  |
| Lymph node involvement |  |  |  |  |  | | |  | |  | |  | |  | |  | |  | | | | |  |  |  |
| N0 (n=57) | 14 (24.6) | 1 (1.8) | 2 (3.5) | 19 (33.3) | 6  (10.5) | | | 22 (38.6) | | 13 (22.8) | | 2 (3.5) | | 10 (17.5) | | 2 (3.5) | | 2  (3.51) | | | | |  |  |  |
| N1-3 (n=45) | 11 (24.4) | 3  (6.7) | 3  (6.7) | 14 (31.1) | 3  (6.7) | | | 16 (35.6) | | 6  (13.3) | | 0 (0.0) | | 7  (15.6) | | 5  (11.1) | | 4  (8.9) | | | | |  |  |  |
| Distance metastasis |  |  |  |  |  | | |  | |  | |  | |  | |  | |  | | | | |  |  |  |
| M0 (n=94) | 24 (25.8) | 4 (4.3) | 5 (5.4) | **28 (29.8)** | | 9  (9.7) | | | 35 (37.6) | | 18 (19.3) | | 2 (2.2) | | 17 (18.3) | | 6 (6.5) | | 5  (5.4) | | | | |  |  |
| M1 (n=10) | 1 (9.1) | 0 (0.0) | 0 (0.0) | **6**  **(60.0) ^d^** | | 0  (0.0) | | | 3 (27.3) | | 1  (9.1) | | 0 (0.0) | | 0  (0.0) | | 1 (9.1) | | 1  (9.1) | | | | |  |  |
| UICC stage |  |  |  |  |  | |  | | |  | |  | |  | |  | | | |  | | |  |  |  |
| I/II (n=53) | 13 (24.5) | 1 (1.9) | 2 (3.8) | 16 (30.2) | 6  (11.3) | | 22 (41.5) | | | 13 (24.5) | | 2 (3.8) | | 10 (18.8) | | **1 (1.9)** | | | | 2  (3.8) | | |  |  |  |
| III/IV (n=51) | 12 (23.5) | 3 (5.9) | 3 (5.9) | 18 (35.29) | 4 (7.8) | | 17 (33.3) | | | 6 (11.76) | | 0 (0.00) | | 7 (13.73) | | **6 (11.8) ^e^** | | | | 4  (7.8) | | |  |  |  |
| Differention degree |  |  |  |  |  | |  | | |  | |  | |  | |  | | | |  | | |  |  |  |
| High (n=8) | 2 (25.0) | 1 (12.5) | 1 (12.5) | **5 (62.50)** | 0 (0.00) | | 2 (25.00) | | | 2 ( 25.00) | | 0 (0.00) | | 1 (12.50) | | 0 (0.00) | | | | 0 (0.00) | | |  |  |  |
| Medium (n=81) | 21 (25.93) | 4 (4.94) | 3 (3.70) | **25 (30.86) ^f^** | 7 (8.64) | | 33 (40.74) | | | 16 (19.75) | | 2 (2.47) | | 13 (16.05) | | 6 (7.40) | | | | 3 (3.70) | | |  |  |  |
| Low (n=14) | 2 (14.29) | 0 (0.00) | 1 (7.14) | 4 (28.57) | 3 (21.43) | | 4 (28.57) | | | 0 (0.00) | | 0 (0.00) | | 3 (21.43) | | 1 (7.14) | | | | 1 (7.14) | | |  |  |  |

a, p= 0.0028 ulcerated CRC vs protruded CRC, OR=0.0704

b, p=0.064 between CRC tumor size, OR=0.3214

c, p=0.096 between T1/T2 and T3/T4 OR=2.4316

d, p=0.053 between M0 and M1, OR= 0.2828

e, p=0.044 between UICC I/II and III/IV, OR= 0.1442

f, p=0.071 High differential CRC vs medium differential CRC,OR= 3.7333

**Supplemental Table S7: Comparison of the presence of MICA alleles in patients with PD-L1 expression**

| Allele | PD-L1 neg. n=21 (%) | | PD-L1 pos. n=3 (%) | χ^2^ |  | P value | | OR (95% CI) | |
| --- | --- | --- | --- | --- | --- | --- | --- | --- | --- |
| *002:01 | 4 (19.05) | 0 (0.00) | |  |  |  | |  | |
| *004 | 0 (0.00) | 0 (0.00) | |  |  |  | |  | |
| *007:01 | 3 (14.29) | 0 (0.00) | |  |  |  | |  | |
| *008 | 8 (38.10) | 1 (33.33) | |  |  |  | |  | |
| *009:01 or *049 | 1 (4.76) | 1 (33.33) | | 2.81 |  | 0.094 | | 0.11 (0.004-2.29) | |
| *010:01 | 6 (28.57) | 2 (66.66) | |  |  |  | |  | |
| *012:01 | 3 (14.29) | 1 (33.33) | |  |  |  | |  | |
| *017 | 1 (4.76) | 0 (0.00) | |  |  |  | |  | |
| *019 | 7 (33.33) | 1 (33.33) | |  |  | |  | |  |
| *027 | 2 (9.52) | 0 (0.00) | |  |  | |  | |  |
| *045 | 2 (9.52) | 0 (0.00) | |  |  | |  | |  |

**Supplemental Table S8:** **Comparison of biomarker CA19-9 to MICA alleles**

| Allele | CA19-9 neg. n=84 (%) | CA19-9 pos. n=15 (%) | χ^2^ | P value | OR (95% CI) |
| --- | --- | --- | --- | --- | --- |
| *002:01 | 20 (23.81) | 3 (20.00) |  |  |  |
| *004 | 4 (4.76) | 0 (0.00) |  |  |  |
| *007:01 | 5 (5.95) | 0 (0.00) |  |  |  |
| *008 | 26 (30.95) | 7 (46.67) |  |  |  |
| *009:01 or *049 | 10 (11.90) | 0 (0.00) |  |  |  |
| *010:01 | 34 (40.48) | 2 (13.33) | 2.96 | 0.077 | 0.23 (0.02-1.11) |
| *012:01 | 15(17.86) | 3 (20.00) |  |  |  |
| *017 | 2 (2.38) | 0 (0.00) |  |  |  |
| *019 | 15 (17.86) | 1 (5.67) |  |  |  |
| *027 | 4 (4.76) | 2 (13.33) |  |  |  |
| *045 | 6 (7.14) | 0 (0.00) |  |  |  |
